# Hybrid task and motion planning with reactive collision handling for multi-robot disassembly of complex products: application to EV batteries

**DOI:** 10.3389/frobt.2026.1857847

**Published:** 2026-07-16

**Authors:** Abdelaziz Shaarawy, Cansu Erdogan, Rustam Stolkin, Alireza Rastegarpanah

**Affiliations:** 1 Extreme Robotics Laboratory, School of Metallurgy and Materials, University of Birmingham, Birmingham, United Kingdom; 2 Department of Applied Artificial Intelligence and Robotics, School of Computer Science and Digital Technologies, Aston University, Birmingham, United Kingdom

**Keywords:** dynamic collision avoidance, learning from demonstrations, multi-robot systems, reactive replanning, task and motion planning

## Abstract

This paper addresses the problem of multi-robot coordination for complex manipulation task sequences. We present a vision-driven task-and-motion planning (TAMP) framework for a real dual-agent platform that integrates task decomposition and allocation with a learning-based planner. A GMM-informed RRT motion planner is coupled with a hybrid safety layer that combines predictive collision checking in a MoveIt/FCL digital twin with reactive avoidance and replanning. This integration is challenging as the system jointly satisfies task precedence, geometric feasibility, dynamic obstacle avoidance, and dual-arm coordination constraints. The framework operates in a closed loop by updating the remaining task sequence from repeated scene scans and completion-state tracking rather than executing a fixed open-loop plan. In EV battery disassembly experiments, compared with baseline RRTConnect under identical perception and task assignments, the proposed system reduces cumulative end-effector path length from 48.8 to 17.9 m (−63.3%) , improves makespan from 467.9 to 429.8 s (−8.1%) , and reduces swept volumes (R1: $0.583\to 0.139\;m^{3}$ , R2: $0.696\to 0.252\;m^{3}$ ) and overlap $\left(0.064\to 0.034\;m^{3}\right)$ . These results show that combining predictive planning and reactive collision avoidance in a real dual-arm disassembly scenario improves motion compactness, safety, and scalability to broader multi-robot sequential manipulation tasks.

## 1 Introduction

Task planning enables autonomous robots to execute complex, multi-step operations under resource constraints by selecting and sequencing actions. When coupled with motion planning, it determines *how* and *when* to act amid environmental dynamics and hardware limits. In single-robot settings, learning- and optimization-based approaches (e.g., ANFIS, GA, RL) improve allocation and ordering under uncertainty (Tejer et al., 2024). In multi-robot systems, continuous coordination, collision constraints, and shared resources make scheduling substantially more challenging. Electric-vehicle (EV) battery disassembly is an especially demanding, safety-critical domain where tasks are sequential and tightly coupled, fixtures and tolerances are strict, and the workspace is cluttered and dynamic (Wegener et al., 2014; Wang et al., 2023). In practice, the main difficulty is not merely combining task planning and motion planning, but making them operate reliably under perception noise, dual-arm coordination complexity, dynamic obstacles, and simultaneous task-level and geometric constraints.

This work presents a perception-driven TAMP architecture for coordinated multi-robot operation in dynamic environments (Figure 2). Perception serves as the entry point to the pipeline and feeds four planning and execution layers: task decomposition, task allocation, offline predictive motion planning, and online reactive execution. Stereo RGB-D sensing with YOLOv8 provides object identities and poses; the task layer enforces precedence, accessibility, and tool-compatibility constraints. The motion layer combines a MoveIt (Coleman et al., 2014)/FCL (Pan et al., 2012) digital twin for predictive collision checking with a reactive avoidance module during execution. In EV battery disassembly, this closed-loop architecture continuously updates both sequences and trajectories from scene feedback, enabling robust dual-robot cooperation rather than a fixed open-loop pipeline.

This paper demonstrates a closed-loop dual-arm system in which planning, perception, and execution remain tightly coupled on real hardware. Building on the GMM-informed RRT planner introduced in Shaarawy et al. (2026), which utilizes a Gaussian Mixture Model (GMM) to guide the sampling region of the Rapidly-exploring Random Tree (RRT) algorithm, this work embeds the planner within a vision-driven dual-arm TAMP framework (Erdogan et al., 2024). The resulting framework continuously handles object-state updates, task-allocation changes, inter-robot interference, and dynamic collision risks while preserving the regulatory disassembly sequence. Hence, the main contributions of this article are as follows:

- Hybrid predictive/reactive collision handling: we combine predictive digital-twin collision checking with reactive avoidance and vision-driven replanning. This coupling supports dual manipulation in dynamic and scalable multi-robot settings.
- Closed-loop coupling between task and motion layers: the system re-scans the scene, tracks object-completion status, and updates the remaining lookup table sequence during execution, rather than treating task planning and motion planning as fully decoupled stages.
- Real-world validation in EV battery disassembly: across the presented three case studies, the integrated framework yields more compact trajectories, lower swept-volume overlap, and improved execution efficiency under real-world uncertainty.

## 2 Related work

Task-and-motion planning (TAMP) is central to multi–robot operation in shared workspaces: it must choose feasible action sequences, allocate limited resources, and coordinate agents while guaranteeing geometric and kinematic safety. The integration of symbolic task planning with continuous motion planning is widely recognized as a key enabler for autonomous execution in complex environments through logic–based representations and discrete decision mechanisms.

### 2.1 Motion planning

Motion planning computes safe, efficient trajectories under kinematic limits and obstacles; in multi-robot settings, it must also coordinate agents for collision-free, synchronized execution. Sampling-based motion planning (SBMP) provides probabilistic completeness and efficient exploration in high-dimensional spaces (Kavraki et al., 1996; LaValle, 1998). Integrating SBMP with TAMP links discrete task decomposition to continuous geometric trajectories and supports dynamic replanning when tasks or preconditions change (Thomason and Knepper, 2019). Empirical studies (e.g., bin-picking) report substantial variation across SBMP variants in time, success, and path quality, motivating task-aware selection and tuning (Realpe et al., 2022). Guided SBMP adapts sampling to real-time perception for on-the-fly obstacle handling (Khanal et al., 2023), while kinodynamic extensions such as iDb-RRT combine motion primitives with optimization to satisfy dynamics and yield smoother, executable paths (Ortiz-Haro et al., 2024).

Learning-based planners target adaptation under uncertainty: deep models show strong performance in dense obstacles with curated datasets (Sarwar et al., 2021), and surveys compare deterministic, heuristic, and learning-based methods across optimality, computation, and robustness (Noroozi et al., 2023). Recent DRL advances coordinate global target sequencing with local obstacle-aware navigation via hierarchy and curriculum/experience reuse, outperforming RRT*/PRM in success rate (Fan, 2023).

Recent manipulator benchmarks further show why baseline choice must be interpreted in context. Astorquia et al. compared sampling-based OMPL planners with a learning-based DRL planner for industrial robotic arms and reported superior performance of the learning-based approach in several key trajectory-quality metrics, including trajectory smoothness and configuration-space length (Astorquia et al., 2025). We build on these findings, together with our earlier evaluation of the GMM-informed planner used in this paper against additional planners (Shaarawy et al., 2026). However, this paper focuses on system-level integration, asynchronous dual-arm execution, perception updates, and reactive inter-robot collision handling on real hardware. We therefore use RRTConnect in MoveIt as the practical baseline because it is the default, widely adopted OMPL planner in many MoveIt deployments and provides a reproducible comparison point for the integrated TAMP system.

Reliable perception underpins both sampling- and learning-based planners. Probabilistic 3D maps such as OctoMap enable dynamic updates for moving obstacles (Hornung et al., 2013). Recent task-conditioned planners also motivate our use of a learned sampling prior: the recently introduced GMM-informed RRT planner in Shaarawy et al. (2026) demonstrated planner-level benefits in constrained manipulation; the present paper studies the distinct problem of integrating that planner into a closed-loop dual-arm TAMP system.

### 2.2 Task planning

Task planning determines action sequences and allocates resources (time, energy, computation) for complex, time-sensitive tasks. In single-robot settings, learning- and optimization-based approaches (e.g., ANFIS, GA, RL) improve ordering and adaptation under uncertainty (Xidias et al., 2021; Tejer et al., 2024). Hybrid models link symbolic reasoning with continuous control (e.g., affordance-guided wayfields) to connect abstract plans to real-time motion in cluttered, human-shared spaces (McMahon et al., 2018). Data-driven methods, notably DRL, enable adaptation but incur heavy training cost and may struggle in highly dynamic scenes (Tejer et al., 2024).

Structured knowledge offers robustness via ontology-based planning that fuses symbolic and geometric reasoning; these methods formalise manipulation tasks and respect spatial constraints but depend on predefined ontologies and can be brittle in unfamiliar environments (Zhao et al., 2018). Moving beyond dyads, scaling to multi-robot teams introduces distribution and synchronization issues. General-purpose planners cover logical constraints yet leave real-time responsiveness under uncertainty less explored (Antão et al., 2022). Heuristic and metaheuristic schedulers (e.g., simulated annealing, dynamic scheduling) trade solution quality for speed (Wang and Chen, 2022; 2024). Optimisation-based formulations (MILP/CP) improve allocation and conflict avoidance, while geometry-aware variants incorporate proximity and subtask dependencies; nevertheless, many approaches face limits in online adaptability and scalability in cluttered workcells (Fatemi-Anaraki et al., 2023; Pan et al., 2021; Motes et al., 2020; Bae et al., 2023).

### 2.3 Integrated TAMP and multi-robot coordination

Integrated TAMP couples symbolic reasoning with geometry-aware motion to satisfy semantic preconditions, spatial constraints, and resource limits in shared workcells (Touzani et al., 2022; Bernardo et al., 2023). Recent efforts move toward distributed, feedback-coupled planning with feasibility-aware distribution, but often assume fixed uncertainty models or analyse failure recovery only partially (Pan et al., 2024; Bouhsain et al., 2024). In multi-robot settings, the main challenge is preserving global task feasibility while accommodating robot-specific reachability, interference, and asynchronous execution. Our work targets this gap by coupling perception updates, task allocation, predictive motion planning, and reactive replanning within one execution loop on real hardware.

### 2.4 Hybrid predictive/reactive collision avoidance

Shared workspaces require both predictive and reactive safety mechanisms. Predictive collision checking through motion-planning frameworks such as MoveIt and proximity libraries such as FCL enables geometric validation before execution (Coleman et al., 2014; Pan et al., 2012), while probabilistic 3D maps such as OctoMap support online updates for moving obstacles (Hornung et al., 2013). However, predictive checking alone is insufficient when obstacles or neighbouring robots move during execution. This motivates hybrid schemes in which a validated plan is continuously monitored and locally corrected or replanned when the scene changes. The present paper contributes such a hybrid predictive/reactive formulation in a real dual-arm disassembly cell, where perception updates, inter-robot distance monitoring, and replanning must operate jointly rather than as isolated modules.

MoveIt 2 Hybrid Planning follows a related principle by combining a slower global planner with a faster local planner that run recurrently with different scopes and communicate through ROS two interfaces (MoveIt 2026). Our architecture is conceptually compatible with this hybrid-planning view, but it is tailored to a ROS 1/MoveIt one dual-arm disassembly cell. The main differences are that each arm keeps an asynchronous planner/executor, both arms share planning-scene awareness through synchronized scene updates, and the global sampling process is biased by TP-GMM demonstrations while inter-robot distances are monitored explicitly during execution.

## 3 System configuration

**FIGURE 1:**
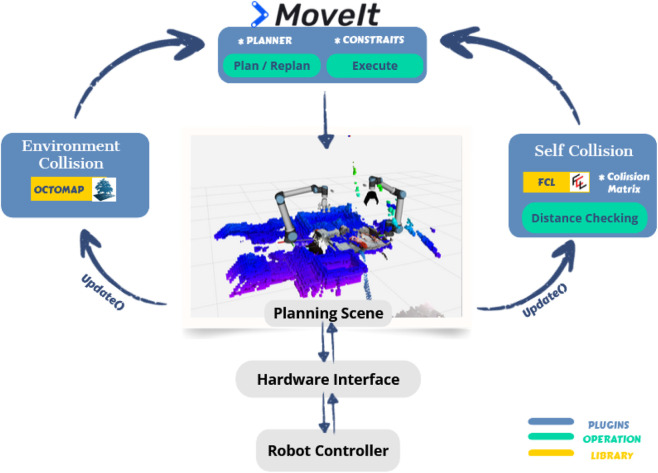
System architecture for predictive planning and reactive execution in MoveIt.

We implement a ROS-based multi-robot stack for safe, coordinated operation in shared workspaces. A MoveIt-based control node orchestrates motion planning, manipulation, trajectory execution, and collision avoidance. The *Planning Scene* aggregates robot kinematics, sensor input, and environment representations into a continuously updated state used by the planner (OMPL) and collision checker (FCL). Figure 1 outlines the architecture and data flow.

*MoveIt and the planning problem:* Planning and replanning are posed as a constrained optimization over robot configurations $q\in C\subset R^{n}$ :

$$\underset{q\in C}{\min}\;f\left(q\right)\;\text{s.t.}\;d\left(q,t\right)\;\geq \;d_{\min},$$

where f(q) encodes path/time/energy costs, and d(q,t) is the *signed distance margin* to the nearest obstacle at time t (positive: safe, zero: contact, negative: penetration). Safety is enforced *along the trajectory*, i.e., $d\left(q\left(s\right),t\right)\geq d_{\min}$ for all path parameters s∈[0,1] .

*Environment collision:* An OctoMap-based 3D occupancy model provides geometry for environment queries. Let $d_{\text{env}}\left(q,t\right)$ be the minimum signed distance from any robot link to mapped obstacles. The constraint

$$d_{\text{env}}\left(q,t\right)\;\geq \;d_{\min}$$

ensures sufficient separation; whenever violated, the system triggers replanning or imposes local motion constraints to restore feasibility.

*Self-collision:* Self-collision is monitored via FCL using a precomputed collision matrix and continuous distance checks between link pairs. With $d_{\text{self}}\left(q\right)$ the minimum inter-link signed distance,

$$d_{\text{self}}\left(q\right)\;\geq \;d_{\min}$$

guarantees collision-free internal configurations during execution.

*Planning Scene (*constraint *aggregation):* The Planning Scene maintains both constraints concurrently; equivalently,

$$d_{\text{total}}\left(q,t\right)\;=\;\min\left\{\;d_{\text{env}}\left(q,t\right),\;d_{\text{self}}\left(q\right)\;\right\}\;\geq \;d_{\min}.$$

This aggregation prevents false safety due to additive cancellation and supports consistent, real-time updates as perception refreshes the map and as robots move. Coordination is implemented as shared-state synchronization rather than strict low-level clock synchronization: each robot runs its own task and motion planner, publishes joint states and scene updates through ROS, and receives the same updated Planning Scene before committing or revising its next trajectory. In this design, asynchronous execution is safe because each robot continuously reasons over the other robot’s most recent state and replans when the shared scene becomes invalid.

*Robot Controller:* The robot controller executes time-parameterised joint trajectories and streams setpoints to the drives at 250 Hz with safety limits, interpolation, and controlled stopping on faults. This controller frequency is distinct from the planning-scene update rate, which is limited by perception and collision-scene processing. Joint states and tracking errors are fed back to the planning stack, closing the loop between planning and physical actuation for asynchronous dual-robot execution.

## 4 Task-and-motion planning (TAMP)

This section presents our task-and-motion planning (TAMP) framework for autonomous multi-robot disassembly of EV battery packs. Implemented in ROS/MoveIt, the system comprises (i) a logic-based task planner that schedules symbolic actions under temporal, spatial, and resource constraints and (ii) a learning-guided motion planner that produces adaptive, collision-free trajectories satisfying geometric and kinematic feasibility. Real-time perception, online replanning, and reactive control enable robust, coordinated, and safe behavior in dynamic, unstructured environments.

### 4.1 Task planner

**FIGURE 2:**
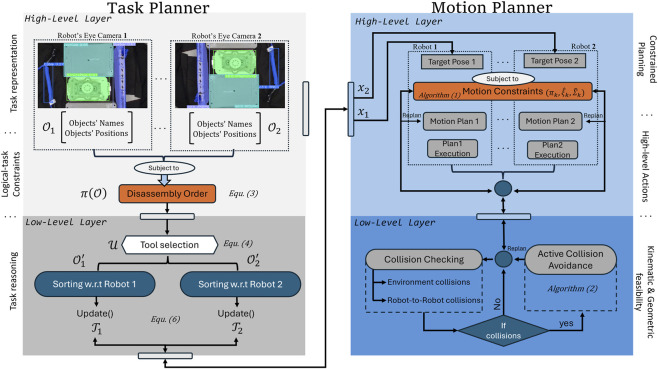
Overview of the proposed TAMP framework in a dual-arm setup. Perception feeds task decomposition, task allocation, offline/predictive planning, and online/reactive execution in a closed loop.

Figure 2 summarizes the perception-driven task-planning stack that coordinates the disassembly with two manipulators. Perception is treated as an explicit front-end rather than as part of the task planner itself: Intel RealSense RGB-D sensing first provides component IDs and 3D pose estimates, which are then consumed by a *task-decomposition* module to enforce precedence and by a *task-allocation* module to assign robots and tools. Execution is monitored in closed loop; after each completed, aborted, or re-planned action, the robots reset to observation poses, the scene is re-scanned, and the remaining task sequence is updated while preserving safety constraints. Hence the task layer is coupled to motion and execution through repeated scene updates rather than operating as a one-shot symbolic plan.

#### 4.1.1 Perception and task representation

The perception pipeline begins with a *task representation* stage, where an Intel RealSense RGB-D camera is integrated with YOLOv8-Seg (Jacob Solawetz, 2024) to detect and segment battery-pack components at the part level. Following the methodology in Erdogan et al. (2024), but using real rather than simulated data, we report detection, segmentation, and 3D localisation metrics under real-world conditions. At the beginning of each execution cycle, and again after each completed or aborted manipulation, the active robot returns to its observation pose so that the current scene state can be verified before the next action is committed. A dataset of 226 real images of NISSAN e-NV200 battery components was collected, covering five object classes: screws, cable, busbar, battery module, and service plug. Of these, 204 images were allocated for training and 22 for validation. Manual labelling and dataset augmentation expanded the dataset to 542 images (474 training, 45 validation, and 23 test). Model training was performed using YOLOv8 on Google Colab (Ubuntu 20.04, Intel Core i7-13700H CPU, NVIDIA GeForce RTX 3050 GPU). Input images were resized to 640×640 pixels, balancing accuracy and computational efficiency. Training was run for 50 epochs and completed in approximately 0.317 h. The final trained model was exported and integrated into the perception stage. The segmentation masks are fused with depth information to recover each component’s 3D centroid and, when required, its 6-DoF pose. These estimates are then provided to the grasping and motion planning modules for execution. After 6-DoF pose acquisition, the information is passed to the *logical-task* stage, where objects are sorted into a predetermined disassembly sequence (Hathaway et al., 2025). This ensures that task reasoning, safety checks, and robot/tool selection are performed in the correct order before execution proceeds.

#### 4.1.2 Multi-robot task scheduling

The *Task Planner* module generates ordered task sequences for each robot, subject to logical and physical constraints. Let

$$O=\left\{o_{1},o_{2},\ldots ,o_{N}\right\}$$ (1)

denote the set of detected disassembly components, where each component $o_{i}$ is represented as

$$o_{i}=\left(label_{i},n,x_{i},u_{i},s_{i}\right),$$ (2)

where $label_{i}$ is the class label, n is the number of detected instances, $x_{i}=\left\{x_{i1},\ldots ,x_{in}\right\}$ is the list of estimated object poses with $x_{in}\in SE\left(3\right)$ , $u_{i}\in U$ is the required tool, and $s_{i}\in \left\{pending,in-progress,completed\right\}$ is the completion state.

Stage 1: Object Detection - At the initial state, and again after each execution update, each robot’s eye-in-hand RGB-D camera acquires a frame from a predefined observation pose and queries a YOLOv8-based detection server. The output is the current unordered set O of detected objects, their 3D poses, and their latest completion states (Equation 1).

Stage 2: Priority-based Sorting - Each object $o_{i}$ is assigned a priority score π(i) by consulting an expert-encoded lookup table that reflects safety, disassembly dependencies, accessibility, and tool constraints, following (Hathaway et al., 2025). This lookup-table design is intentional: for EV battery disassembly, regulatory procedure and safety compliance are better expressed as hard expert rules than as soft costs inside a generic symbolic search. The table therefore encodes the abstract task structure of the process, while remaining extensible–new object classes or task types can be incorporated by adding new rows with precedence, tool, and safety annotations. Objects are then ordered according to the predefined disassembly sequence π ,

$$O^{\prime }=\pi \left(O\right)$$ (3)

ensuring logical ordering (e.g., screws ≺ battery module), consistent with prior robotic EV-battery dismantling studies showing that automated fastener or nut removal is a key enabling step before module separation (Rastegarpanah et al., 2021b). Table 1 illustrates the mapping between priority scores, battery components, and required tools.

**TABLE 1:** Expert-encoded lookup table for EV battery disassembly, mapping component classes to precedence priority and required tool.

Priority score (π)	Component	Required tool
1	Screws	3F gripper
2	Battery module	Vacuum gripper
3	Service plug	3F gripper
4	Busbar	3F gripper
5	Cable	3F gripper

Stage 3: Tool-based Assignment - Let $R=\left\{R_{1},R_{2}\right\}$ denote the set of robots, each equipped with a distinct tool set $U_{j}\subseteq U$ . The prioritized task list $O^{\prime }$ (Equation 3) is partitioned into subsets

$$O_{j}^{\prime }=\left\{o_{i}\in O^{\prime }|u_{i}\in U_{j}\right\},$$ (4)

such that each task $o_{i}$ is assigned to the robot $R_{j}$ capable of executing it.

Stage 4: Cost-based Sorting - After logical ordering and tool assignment, objects in each subset are further sorted according to an execution cost function $J_{j}$ . The cost is used as a practical metric for relative task ordering and allocation in SE(3) . In generalized form, a pose-distance heuristic includes both translation and orientation. For a pose $x_{i}=\left(R_{i},t_{i}\right)\in SE\left(3\right)$ and the robot end-effector pose $p=\left(R_{p},t_{p}\right)\in SE\left(3\right)$ , we write

$$d\left(x_{i},p\right)=w_{t}\;\|t_{i}-t_{p}{\|}_{2}+w_{R}\;e_{R}\left(R_{i},R_{p}\right),$$ (5)

where $e_{R}\left(R_{i},R_{p}\right)$ denotes an orientation-error term and $w_{t},w_{R}\geq 0$ balance translational and rotational influence. In the experiments reported here, task ordering is dominated by the relative Cartesian positions of the objects and robot end effectors; therefore, we use this expression primarily through its translational component by setting $w_{t}=1$ and using a small or zero rotational weight $w_{R}$ . This is sufficient for selecting nearby tasks while preserving precedence and tool constraints.

The final ordered task sequence for robot $R_{j}$ is then obtained by minimizing the accumulated heuristic cost over feasible task orderings,

$$T_{j}=\arg\underset{\sigma_{j}\in \Pi \left(O_{j}^{\prime }\right)}{\min}\overset{\left|O_{j}^{\prime }\right|}{\underset{m=1}{\sum }}d\;\left(x_{\sigma_{j}\left(m\right)},p_{j}^{\left(m-1\right)}\right),$$ (6)

where $\Pi \left(O_{j}^{\prime }\right)$ denotes the set of feasible orderings after enforcing precedence and tool constraints (Equation 4), $\sigma_{j}$ is one such ordering, $p_{j}^{\left(m-1\right)}$ is the robot pose after the previous task, and $p_{j}^{\left(0\right)}$ is the current end-effector pose. Finally, the Task Planner outputs

$$T=\left\{T_{1},T_{2}\right\},$$

where each $T_{j}$ is an optimized task sequence for robot $R_{j}$ . These sequences respect (i) disassembly precedence, (ii) tool compatibility, and (iii) translation-dominant pose-distance efficiency, and are passed to the *Motion Planner* for trajectory generation.

Closed-Loop Task-State Update - After each pick, place, or interruption event, the robots reset to their observation poses and re-scan the workspace to verify the current object states. If an item has been successfully removed from its source and confirmed at its destination, its completion state is set to completed; otherwise it remains pending or is returned to the queue for replanning. The lookup table ordering is then updated by removing completed objects and re-sorting the remaining items according to the refreshed scene, accessibility, and tool constraints. Consequently, task decomposition and task allocation are updated online as the motion layer changes the scene, showing that the two layers are coupled in closed loop rather than fully decoupled.

### 4.2 Predictive motion planning and reactive execution

In this section, we describe the motion component of the proposed TAMP framework for multi-robot setups. For each robot $R_{j}$ , the planner receives the optimized task sequence $T_{j}$ from the task layer (Equation 6) together with the latest scene state. The corresponding object poses $x_{i}$ (from Equation 2) are extracted and transformed from the camera coordinate frame into the robot’s reference frame. Based on these task targets, the motion layer computes feasible trajectories by addressing two key requirements: (i) offline/predictive path generation, achieved via a Learning-from-Demonstration (LfD) approach that biases the sampling process toward expert-like trajectories, and (ii) online/reactive collision handling, ensuring safe execution in multi-robot and cluttered environments.

#### 4.2.1 LfD-guided motion planning

As demonstrated in Shaarawy et al. (2026), integrating Learning-from-Demonstration (LfD) techniques with sampling-based motion planning algorithms leads to highly efficient motion planners. That prior work reported the planner-level comparisons and algorithmic evaluation of the GMM-informed RRT module itself; the present paper therefore builds on that foundation and focuses on integrating the validated planner into a vision-driven dual-arm TAMP system with closed-loop task updates and reactive collision handling. The motion-planning pipeline begins with the collection of demonstrations. Following the approach in Hathaway et al. (2023), demonstrations were recorded for a set of disassembly tasks, extended in this work to a dual-robot setup. In these sessions, one robot executed random motions within the shared workspace, while the other was teleoperated by a human expert. The demonstrations encode trajectories from start to goal poses while avoiding collisions with the other robot. These data capture expert approaches for safe and efficient manipulation in multi-robot environments and are subsequently used to train a probabilistic model that generalizes the underlying motion patterns of both robots.

TP-GMM Learning and Reproduction - Gaussian Mixture Models (GMMs) are employed to capture the variability of demonstrated trajectories in Cartesian space, representing motion data as a weighted sum of K Gaussian components. Model parameters are estimated using the Expectation-Maximisation (EM) algorithm, enabling probabilistic generalisation of expert demonstrations. However, a standard GMM is restricted to a fixed reference frame, which limits its adaptability to changing task conditions. Task-Parameterized GMMs (TP-GMMs) overcome this limitation by encoding demonstrations with respect to multiple task-dependent frames. Following the approach in Shaarawy et al. (2026), these frames are denoted as the start pose ${\hat{f}}^{\left(s\right)}$ , goal pose ${\hat{f}}^{\left(g\right)}$ , and obstacle poses ${\hat{f}}^{\left(o\right)}$ , corresponding to $p_{k}$ and $x_{ik}$ Equation 5, all expressed relative to the base frame of robot $R_{j}$ . Since this work extends (Shaarawy et al., 2026) to a dual-robot setup, for each defined task (e.g., pick or place) two TP-GMMs are learned, one per robot. Now each task demonstration is expressed in the corresponding frames, yielding three GMMs that are subsequently combined. During reproduction, new task parameters are provided, and the model adapts its Gaussian components as follows:

$$\begin{array}{cc} {\hat{\xi }}_{t,k}^{\left(s\right)} & ={\hat{A}}_{t}^{\left(s\right)}\;\mu_{k}^{\left(s\right)}+{\hat{b}}_{t}^{\left(s\right)},\;{\hat{\Sigma }}_{t,k}^{\left(s\right)}={\hat{A}}_{t}^{\left(s\right)}\;\Sigma_{k}^{\left(s\right)}\;{\left({\hat{A}}_{t}^{\left(s\right)}\right)}^{T}\\ {\hat{\xi }}_{t,k}^{\left(g\right)} & ={\hat{A}}_{t}^{\left(g\right)}\;\mu_{k}^{\left(g\right)}+{\hat{b}}_{t}^{\left(g\right)},\;{\hat{\Sigma }}_{t,k}^{\left(g\right)}={\hat{A}}_{t}^{\left(g\right)}\;\Sigma_{k}^{\left(g\right)}\;{\left({\hat{A}}_{t}^{\left(g\right)}\right)}^{T}\\ {\hat{\xi }}_{t,k}^{\left(o\right)} & ={\hat{A}}_{t}^{\left(o\right)}\;\mu_{k}^{\left(o\right)}+{\hat{b}}_{t}^{\left(o\right)},\;{\hat{\Sigma }}_{t,k}^{\left(o\right)}={\hat{A}}_{t}^{\left(o\right)}\;\Sigma_{k}^{\left(o\right)}\;{\left({\hat{A}}_{t}^{\left(o\right)}\right)}^{T} \end{array}$$ (7)

The number of Gaussian components is set to K=5 , as determined by the Bayesian Information Criterion (BIC) in Shaarawy et al. (2026). This produces an adapted GMM with updated parameters ${\left\{\pi_{k},{\hat{\xi }}_{t,k},{\hat{\Sigma }}_{t,k}\right\}}_{k=1}^{K}$ , which generates trajectories consistent with the demonstrated behaviors while conditioned on the new task configuration.

GMM-informed Planner - Our planning framework integrates task space and configuration space. Task space, a subset of SE(3) , represents object poses, grasping poses, and manipulation goals, while configuration space $C\subseteq R^{n}$ handles the robot’s joint states. The TP-GMM is trained and reproduced in task space. It encodes expert demonstrations relative to task-parameter frames $\left({\hat{f}}^{\left(s\right)},{\hat{f}}^{\left(g\right)},{\hat{f}}^{\left(o\right)}\right)$ and produces an adapted distribution ${\left\{\pi_{k},{\hat{\xi }}_{t,k},{\hat{\Sigma }}_{t,k}\right\}}_{k=1}^{K}$ . This defines a high-likelihood region Q in Cartesian space, which is enforced as a constraint during planning. Path generation is performed using the RRTConnect (also referred to as Bi-RRT) algorithm from OMPL (Şucan et al., 2012), where $q_{s}$ (the robot’s current configuration) initializes the start tree $Tree_{s}$ , and $q_{g}$ is computed via inverse kinematics of ${\hat{f}}^{\left(g\right)}$ . At each iteration, a candidate configuration $q_{\mathrm{sample}}\in C$ is drawn. Through rejection sampling, only configurations whose forward kinematics satisfy $FK\left(q_{\mathrm{sample}}\right)\in Q$ are retained. This biases exploration toward task-relevant regions, guiding the planner toward expert-like motions. Algorithm one outlines the GMM-constrained RRTConnect introduced in Shaarawy et al. (2026). The validated samples are used to extend the bidirectional trees, and once connected, a feasible path X∈Q is returned. The resulting trajectory X is a valid, collision-free solution that accomplishes the primary pick-object task while satisfying configuration-space (kinematic) constraints, remaining consistent with expert demonstrations, and satisfying task-space constraints.

Algorithm 1. GMM-informed Bi-RRT.

**Require:** Start, ${\hat{f}}^{\left(s\right)}$ , Goal ${\hat{f}}^{\left(g\right)}$ , and Obstacle ${\hat{f}}^{\left(o\right)}$ task pose frames
**Ensure:** A feasible Cartesian path X from ${\hat{f}}^{\left(s\right)}$ to ${\hat{f}}^{\left(g\right)}$ : X∈Q

1: Q← reproduceGMM ( ${\hat{f}}^{\left(s\right)}$ , ${\hat{f}}^{\left(g\right)}$ , ${\hat{f}}^{\left(o\right)}$ ) ⊳ (Equation 7)
2: $q_{s}\leftarrow q_{\mathrm{current}}$ (robot’s current configuration)
3: $q_{g}\leftarrow IK\left({\hat{f}}^{\left(g\right)}\right)$

4: $Tree_{s}$ InitTree $\left(q_{\text{s}}\right)$

5: $Tree_{g}$ InitTree $\left(q_{\text{g}}\right)$

6: **while** not Terminated () **do**
7: $q_{\text{sample}}\leftarrow$ SampleConfiguration (Q)

8: $q_{\text{new}}\leftarrow$ extendtree ( $Tree_{s}$ , $q_{\text{sample}}$ )
9: **if** $q_{\text{new}}\neq$ None **then**
10:  **if** connecttree ( $Tree_{g},q_{\text{new}}$ ) **then**
11:   **return** extractpath ( $Tree_{s}$ , $Tree_{g}$ )
12:  **end if**
13: **end if**
14: swap ( $Tree_{s}$ , $Tree_{g}$ )
15: **end while**
16: **return** failure

#### 4.2.2 Reactive Collision Monitoring and avoidance

In addition to path optimization through LfD, the second critical component of our motion planner is *collision perception and avoidance*, which ensures safe execution of trajectories in a shared dual-arm workspace. We investigate two UR10e manipulators operating in proximity, where inter-robot collisions and interactions with dynamic obstacles are key challenges. To address this, the proposed Active Collision Avoidance module combines real-time perception with avoidance strategies, structured into two complementary parts: *Perception* and *Avoidance*.

##### 4.2.2.1 Collision perception

Collision detection is handled through the Flexible Collision Library (FCL) within a MoveIt Planning Scene. All collision objects, including the two robots (from their URDFs) and the environment, are monitored in real time.

**FIGURE 3:**
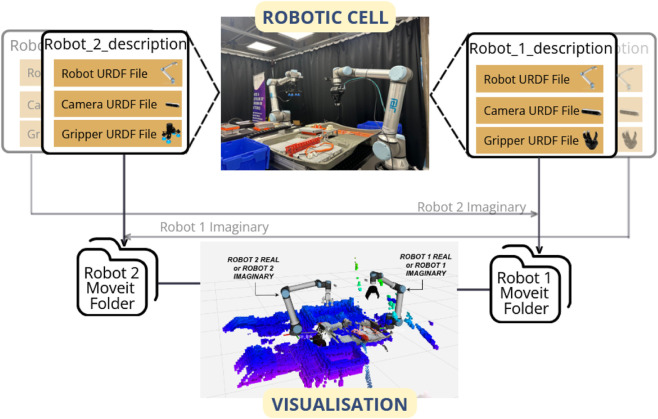
Integration of real and virtual (imaginary) robots in a shared simulation, where each URDF embeds the counterpart’s model to enable inter-robot collision checking through FCL.

Robot-To-Robot collisions - MoveIt’s Allowed Collision Matrix (ACM) is used to encode feasible body interactions. Since dual-robot systems require bidirectional awareness, we employ a digital twin strategy: each URDF embeds a replica of the other robot (Figure 3), enabling independent but coordinated collision checking. The ACM is further refined by excluding irrelevant body pairs to reduce computational load.

Environment collisions - An Intel RealSense depth camera provides dynamic 3D workspace perception via MoveIt’s Occupancy Map Updater, which convert depth data into a voxel-based OctoMap with 0.03 m resolution. This OctoMap is broadcast through ROS shared-scene updates to both robots’ planning scenes, allowing each robot to reason about environmental and inter-robot constraints from a consistent state estimate. Due to the computational demands of maintaining two planning scenes, the maximum achievable planning-scene update rate is ∼ 10 Hz. No explicit low-level clock synchronization is imposed between the two manipulators; instead, coordination relies on these shared-state updates, current joint-state feedback, and continuous dynamic collision monitoring.

**FIGURE 4:**
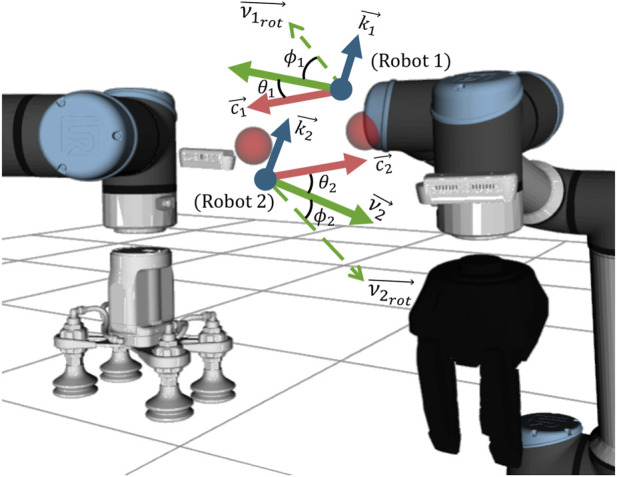
Reactive avoid_collision() routine triggered when two robots approach the early-warning threshold ε . The red spheres denote the contact pair reported by FCL, after which the controller locally steers away and requests replanning.

A safety threshold ε=0.15 m is defined to detect near-collisions, with violations triggering reactive adjustments to increase separation. This corresponds to five OctoMap voxels and was chosen as a conservative early-warning margin that provides reaction distance at the available update rate while avoiding excessive false triggers. Limiting collision checks to distances ≤ε reduces planning complexity. When a proximity violation is detected, FCL returns 3D contact points between objects, which are visualized in RViz (visualization_msgs/MarkerArray) as shown in Figure 4.

Algorithm 2. Reactive Collision Monitoring and Replanning.

1: Initialize planning-scene monitor and collision/distance services
2: **while** system active **do**
3: Update planning scene from sensor and robot-state feedback
4: Query collision state and minimum distance
5: **if** collision detected **then**
6:  Stop current trajectory execution
7: **else if** minimum distance ≤ε **then**
8:  Invoke avoid_collision () and request replan ()
9: **end if**
10: Publish collision and near-collision markers
11: **end** while

##### 4.2.2.2 Collision avoidance

Collision avoidance is implemented through a dedicated avoid_collision() routine, enabling both robots to adapt dynamically to near-collisions in real time. The approach employs a velocity vector steering strategy in SO(3) , where the robot’s tool velocity vector $\vec{v}$ is reoriented when a collision vector $\vec{c}=\sum_{n=1}^{N}\vec{c_{n}}$ (aggregated from detected near-collisions) indicates a potential obstacle (Figure 4). The steering axis is computed as $\vec{k}=\vec{c}\times \vec{v}$ , and Rodrigues’ rotation formula is applied to yield a rotated velocity vector ${\vec{v}}_{\text{rot}}$ .

$${\vec{v}}_{\mathrm{rot}}=\vec{v}+sin\left(\phi \right)\cdot \left(\vec{k}\times \vec{v}\right)+\left(1-cos\left(\phi \right)\right)\cdot \vec{k}\times \left(\vec{k}\times \vec{v}\right)$$ (8)

A steering angle ϕ regulates the correction magnitude. Rather than fixing ϕ , we define it to be relative to the angle θ between $\vec{c}$ and $\vec{v}$ :

$$\theta =\cos^{-1}\left(\frac{\vec{c}\cdot \vec{v}}{\left\|\vec{c}\|\|\vec{v}\right\|}\right),\;\phi =\frac{\pi }{2}-\theta ,$$ (9)

so that imminent collisions (small θ ) yield larger steering corrections, while tangential encounters (large θ ) produce minimal deviations.

This adaptive behavior avoids unnecessary trajectory deviations, helping to maintain path optimality while ensuring safety through timely avoidance maneuvers. Algorithm two presents the overall Active Collision Avoidance component in the motion planner, with the avoid_collision() routine being invoked when near-collision state is flagged to interrupt the robot controller’s execution of the current planned trajectory. The routine remains active until the near-collision state clears. Each robot then requests replan() (Algorithm 1) to generate a collision-free trajectory from its current configuration to the target pose. The active collision-avoidance algorithm runs iteratively until the robot converges to the target pose.

### 4.3 Framework overview and information flow (dual-arm setup)

Figure 2 illustrates the proposed TAMP framework implemented on a dual-arm robotic setup, asynchronously controlling two robots to complete a full disassembly task. The framework is organized as a perception front-end plus four planning/execution layers, ensuring both long-horizon task feasibility and safe, coordinated dual-arm manipulation. The information flow proceeds as follows:

- Perception Front-End: Two eye-in-hand cameras mounted on the robots acquire RGB-D images of the shared workspace. Each camera detects relevant objects, yielding their names, six-dimensional poses, and current completion states, which are passed into the planning stack.
- Task Decomposition: The current scene state is mapped to an abstract disassembly state that respects precedence constraints and the expert lookup table.
- Task Allocation: Feasible operations are assigned to each robot according to tool compatibility, reachability, and execution cost.
- Offline/Predictive Motion Planning: Task allocations are translated into motion-planning goals for each robot, and the GMM-informed RRT planner generates predictive motion plans validated against the digital twin before execution.
- Online/Reactive Execution: The execution layer ensures safe motion through collision monitoring, active avoidance, and replanning. When a near-collision is detected, the avoid_collision() routine is invoked (Equations 8, 9), followed by replanning from the current robot configuration to the target pose. After each action, the robots re-scan the scene before the next task is confirmed.

Therefore, the complete TAMP pipeline operates as a hierarchical loop:

1. Perception update → task decomposition computes the current feasible disassembly order.
2. Task allocation → predictive motion planning generates task-constrained trajectories.
3. Reactive execution → if a proximity violation occurs, invoke active avoidance and replan.
4. Re-scan the scene, update completion states, and repeat until all disassembly goals are achieved.

## 5 Experimental study

Three case studies were conducted to evaluate the proposed multi-robot Task and Motion Planning (TAMP) framework. These are referred to as *Case Study I*, *Case Study II*, and *Case Study III*. *Case Study I* investigates robot-to-robot interactions, focusing on potential collisions and the robots’ ability to replan trajectories while executing tasks in a shared workspace. *Case Study II* evaluates robot performance in the presence of both static and dynamic obstacles. Finally, *Case Study III* integrates both aspects into a real-world application: EV battery disassembly, analyzing coordination, and executing paths. Collectively, these case studies validate the robustness of the proposed approach under diverse operational conditions.

**FIGURE 7:**
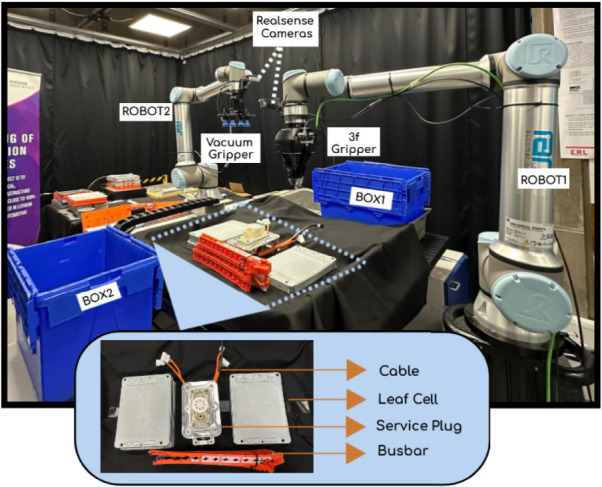
The workspace with a battery pack disassembly setup, equipped with two collaborative robot arms with different end-effectors (a vacuum gripper and a 3F gripper) for manipulation tasks and three depth cameras (two cameras for perception and one camera for collision avoidance).

System Calibration - All experiments were carried out using two UR10e cobots calibrated to a fixed world frame. Each robot was equipped with an eye-in-hand Intel RealSense camera, and an additional eye-to-hand camera was fixed in the workspace. The robots used distinct end-effector tools (Robot 1: 3-fingered gripper; Robot 2: vacuum gripper) as shown in Figure 7. The framework relies on a digital twin with continuous simulation-to-real and real-to-simulation synchronization so that planned trajectories can be executed on hardware while sensor feedback updates the scene model.

Software and Compute Platform - The implementation uses ROS 1, MoveIt 1, and OMPL through the MoveIt planning interface. Perception, task planning, motion planning, collision monitoring, and robot-control interfaces run on a single Linux workstation equipped with an Intel i7 CPU and an NVIDIA 1080 Ti GPU; RAM was shared by the perception, planning, and control processes on the same workstation. Running all modules on one workstation avoids network-distributed timing effects; the dominant synchronization assumptions are therefore the ROS message-passing latency within the workstation and the ∼ 10 Hz shared planning-scene update rate reported above.

### 5.1 Case study I

**FIGURE 5:**
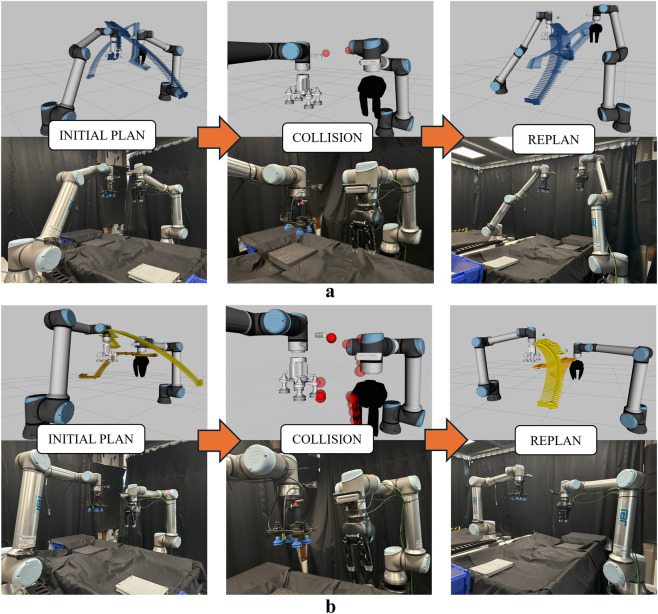
Case study I Robot-to-robot interaction. Comparison between **(a)** baseline RRTConnect and **(b)** GMM-informed RRTConnect in terms of planned-path quality. The figure displays the sequence in which an initial plan encounters a near-collision, triggering replanning that enables both robots to successfully reach their goal poses.

*Case Study I* evaluates the reliability of the motion layer within the TAMP framework in a dual-arm setup. Each robot is assigned a set of target poses corresponding to task allocations defined by the task layer. The focus is on comparing the LfD-guided GMM-informed RRTConnect (Section 4.2.1) against the baseline uniform-sampling RRTConnect planner. The objective is to assess the efficiency of both planners in generating feasible and optimal trajectories while avoiding robot-to-robot collisions in a shared workspace. Figure 5 illustrates the evaluation scenario, where both planners handle situations with potential inter-robot collisions. The study highlights each planner’s capacity to adapt to unpredictable workspace interactions and maintain safe navigation.

### 5.2 Case study II

*Case Study II* evaluates the robots’ collision avoidance capabilities in environments with dynamic obstacles. The experimental setup comprises two UR10e robots performing collaborative tasks. To emulate dynamic disturbances, an object was attached to Robot 1’s end-effector and moved randomly within the workspace, while Robot two executed a transport task: picking up an object, carrying it, and placing it in a designated target area.

**FIGURE 6:**
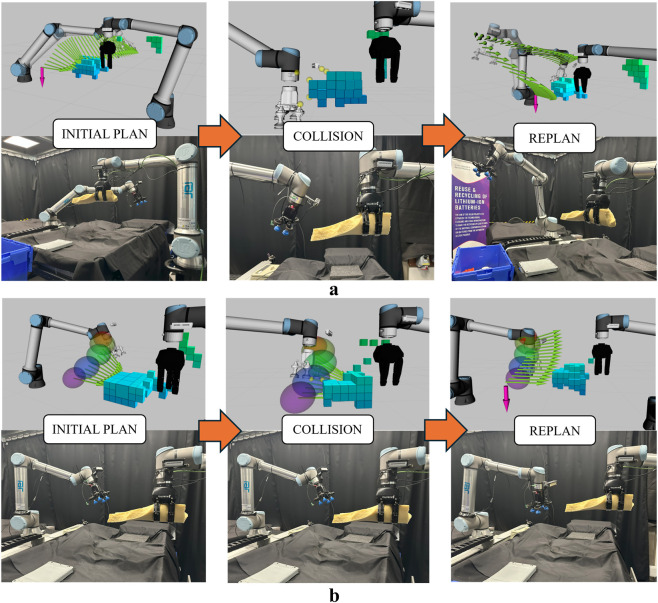
Case study II: Robot-to-dynamic environment interaction. Comparison between **(a)** baseline RRTConnect and **(b)** GMM-informed RRTConnect in terms of path quality. The figure illustrates the planning sequence where an initial plan encounters a near-collision with a dynamic object (in this case, attached to the other robot), triggering replanning that enables the robot arm to successfully reach its goal pose.

Figure 6 shows the test scenario comparing baseline RRTConnect and GMM-informed RRTConnect. The sequence proceeds as follows: (Step 1) Robot two follows its initial plan toward the goal, (Step 2) a near-collision occurs due to Robot 1’s random motion, and (Step 3) Robot two invokes dynamic replanning to generate an alternative, collision-free path. This process ensures safe task completion while adapting to the dynamic environment. The study demonstrates how the proposed planner effectively handles robot-to-dynamic-environment interactions by eliminating collision risks while preserving task execution.

### 5.3 Case study III

This case study presents a realistic disassembly scenario in which two UR10e robots collaboratively dismantle components of a NISSAN e-NV200 electric vehicle battery pack. The robots are equipped with distinct end-effectors ( $u_{1}$ : vacuum gripper, $u_{2}$ : 3-finger gripper) and are assigned complementary tasks accordingly. The disassembly process involves removing key components such as battery modules, cables, busbars, and a service plug (Figure 7). Robots operate without prior knowledge of object locations and rely exclusively on vision-based perception, as in our prior work on robotic cutting (Rastegarpanah et al., 2021a).

The proposed TAMP framework (Figure 2) is executed here as a closed loop. Perception first localizes battery components and confirms their current completion states. Task decomposition and task allocation then generate the current robot-specific action lists according to disassembly order π(O) , tool requirements, and distance-based cost Equation 5. Specifically, robot $R_{1}$ , equipped with the 3-finger gripper, manipulates components such as screws, cables, busbars, and service plugs, while robot $R_{2}$ , fitted with the vacuum gripper, handles battery modules. This complementary tool setup enables flexible handling of heterogeneous component geometries while maintaining the expert-defined disassembly order.

The motion layer integrates four TP-GMM models: two for the pick phase (one per robot) and two for the place phase. Each model is trained, as described in Section 4.2.1, using demonstrations tailored to the respective robot and manipulation stage. During task execution, the robots query the TP-GMM server, which returns the appropriate model for the corresponding robot and stage (pick or place). Collision avoidance is embedded within the control strategy. Both robots continuously monitor their shared workspace to detect potential collisions with each other, as well as with static or dynamic objects. In the event of a collision risk, dynamic replanning is triggered (Algorithm 2), after which the robots re-scan the workspace and update the remaining task list before committing the next action. The framework executes iteratively until the disassembly sequence is fully completed.

### 5.4 Comparison metrics

Each case study was evaluated under both planners, baseline RRTConnect and the GMM-informed planner, using Path Length and Path Duration. Study-specific metrics included the Manipulability Index (Case Study II) and Swept volume (Case Study III). Path length is computed as the cumulative Euclidean distance of end-effector positions along the trajectory between the specified start and end. Reported time metrics denote end-to-end elapsed time from task confirmation in the current scan to successful completion of the corresponding motion. For the proposed planner, this therefore includes TP-GMM reproduction, constrained motion planning, physical execution, and any delays introduced by reactive avoidance/replanning. We report this integrated timing because the contribution of the paper is the coupled real-world system rather than an isolated planner micro-benchmark. Trajectories and cumulative-distance plots are reported for qualitative inspection.

In Case Study II, we additionally evaluate the manipulability index, a configuration-dependent measure of dexterity. It quantifies the arm’s ability to generate end-effector motion across directions, enabling comparison of motion planners and highlighting proximity to singular configurations, particularly informative in cluttered or dynamic scenes. We compute the index following Yoshikawa’s formulation (Togai, 1986). In Case Study II, manipulability is evaluated using Yoshikawa’s index, denoted w (Togai, 1986). Joint and end-effector velocities satisfy $\dot{x}=J\left(q\right)\dot{q}$ where $\dot{x}$ , J(q) , and $\dot{q}$ denote the end-effector velocity, the Jacobian at configuration q , and joint velocities, respectively. To characterise directional dexterity, we analyse the eigenvalues of $JJ^{T}$ and report the condition number

$$w_{\lambda }=\frac{\lambda_{\max}}{\lambda_{\min}},$$ (10)

with larger values indicating proximity to singular configurations, thus loss of dexterity.

In Case Study III, we evaluate the swept volume to quantify the manipulator’s spatial footprint, the 3D region traversed by the links and end-effector over the trajectory. This metric is pertinent in multi-robot or human–robot settings, where lower swept volume implies reduced collision risk and better workspace utilisation. We compute it via a voxel-occupancy model using the same 0.03 m voxel size as the OctoMap collision representation, i.e., the workspace is discretised into cubes of edge h=0.03 m , and the total volume is

$$V=N_{\text{occ}}\;h^{3},$$ (11)

with $N_{\text{occ}}$ the number of voxels occupied during motion. This provides a consistent basis for comparing planners in terms of spatial efficiency. Throughout all case studies, the near-collision trigger is fixed at ε=0.15 m, i.e., five occupancy cells, which provides a conservative but practical early-warning margin for reactive execution.

## 6 Results and discussion

This section reports the quantitative outcomes of the three case studies introduced in Section 5, with emphasis on the contribution-specific metrics of trajectory compactness, reactive safety, and end-to-end coordination quality (Equations 10, 11). 

### 6.1 Case study I: robot-to-robot collision avoidance

**FIGURE 8:**
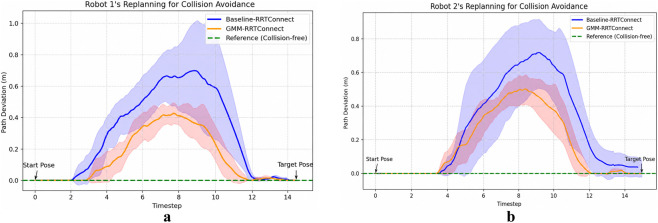
Comparison of path deviations due to collisions detected during motion execution of both robots. **(a)** $R_{1}$ path deviation. **(b)** $R_{2}$ path deviation.

In this case study, we evaluate collision avoidance and re-planning capabilities when two robots operate within a shared workspace. Both robots start from distinct initial poses and move toward predefined targets, creating the potential for inter-robot collisions. The comparison focuses on the deviation from a reference collision-free trajectory, thereby quantifying the quality of the trajectory each planner produces (Figure 8).

Figure 8a shows the deviation profiles of $R_{1}$ . With baseline RRTConnect (blue line), $R_{1}$ reaches a peak mean deviation of 0.7 m , indicating substantial divergence from the collision-free reference. By contrast, the GMM-informed RRTConnect planner (orange line) maintains significantly lower deviations, peaking at approximately 0.4 m . This reduction of nearly 45% highlights the stabilizing influence of the learning-informed prior. Similarly, for $R_{2}$ (Figure 8b), baseline RRTConnect produces deviations exceeding 0.8 m , while GMM-informed RRTConnect consistently remains below 0.6 m . Across the trajectories, the shaded regions (variance bands) further highlight the robustness of the GMM-informed RRTConnect. In particular, baseline RRTConnect exhibits larger variability, with standard deviations of approximately 0.3 for $R_{1}$ and 0.25 for $R_{2}$ , whereas the GMM-informed RRTConnect maintains a maximum standard deviation of only 0.1. This reduction in variability indicates more consistent and reliable replanning performance. Both robots converge back to near-zero deviation when reaching their targets, confirming successful task completion. However, the difference lies in the intermediate trajectories: baseline RRTConnect tends to produce abrupt detours with higher variability, whereas GMM-informed RRTConnect achieves smoother and more predictable paths closer to the reference trajectory (green dashed line).

### 6.2 Case study II: dynamic environment evaluation

This case study examines the comparative performance of the baseline RRTConnect and the proposed GMM-informed RRTConnect planners in a dynamic task execution scenario. The evaluation considers three main aspects: (i) motion planning and execution time, (ii) Cartesian path length, and (iii) manipulability index along the executed trajectories. The goal is to assess the efficiency, consistency, and adaptability of the GMM-informed planner relative to the baseline.

**FIGURE 9:**
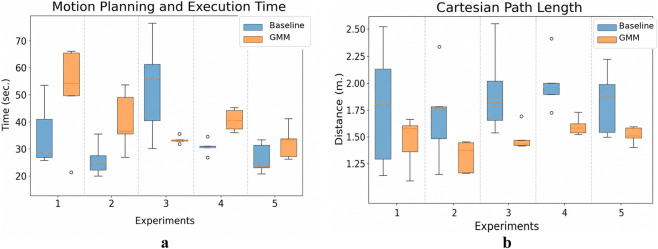
Comparison of baseline RRTConnect and the GMM-informed planner in terms of **(a)** end-to-end motion planning and execution time, and **(b)** Cartesian path length.

Figure 9 presents the experimental outcomes for two of these performance indicators across five independent experiments, each repeated 5 times. Figure 9a illustrates motion planning and execution times, while Figure 9b depicts the resulting Cartesian path lengths. In terms of execution time (Figure 9a), the GMM-informed planner generally requires more time than the baseline RRTConnect. This additional time is due to the reproduction step of the TP-GMM, which generates an expert-informed trajectory in task space once queried with new task parameters (start, goal, and obstacle poses), as described in Equation 7. This reproduced trajectory is then imposed as a constraint in the sampling process, biasing the planner toward expert-like solutions but at the cost of added computation. Despite this increase in planning time, the observed trade-off is justified by the improved quality of the generated trajectories. When comparing Cartesian path lengths (Figure 9b), the GMM-informed planner consistently produces shorter and more efficient paths across all experiments. The baseline RRTConnect, on the other hand, often yields longer and more variable paths, reflecting its tendency to explore larger portions of the configuration space without the benefit of task-informed guidance. The reduced variance in path length observed for the GMM-informed planner further highlights its robustness and consistency in dynamic settings. These results indicate that while the GMM-informed planner introduces a moderate computational overhead, it offers significant improvements in trajectory optimality and reliability; two critical aspects in dynamic, multi-robot disassembly scenarios.

**FIGURE 10:**
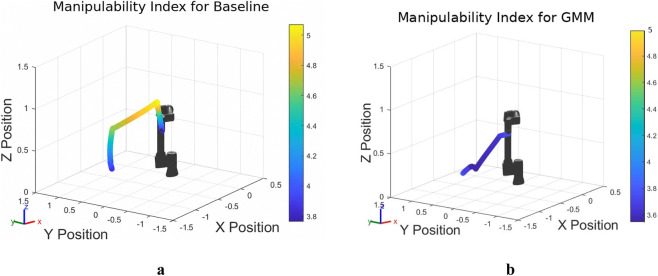
Comparison of manipulability index for two trajectory solutions produced with **(a)** baseline RRTConnect and **(b)** the GMM-informed planner.

Moreover, as shown in Figure 10a, a baseline RRTConnect trajectory exhibits noticeable spikes in the condition-number-based manipulability metric (Equation 10). These spikes indicate that the robot passes close to singular configurations, where joint mobility becomes restricted and control may be less stable. Conversely, a trajectory generated by the GMM-informed planner (Figure 10b) demonstrates a more consistent manipulability profile with lower variance, avoiding abrupt changes that could compromise motion stability. Overall, while the baseline planner occasionally attains locally favourable postures, it does so at the risk of approaching singular configurations. In contrast, the GMM-informed planner produces smoother, more reliable trajectories that better preserve dexterity, making it a safer choice for dynamic and sensitive multi-robot tasks.

### 6.3 Case study III: comparative evaluation of GMM-informed and baseline planners in robotic disassembly

Two UR10e robots perform a collaborative EV-battery disassembly sequence in a shared cell (workspace and cell layout in Figure 7). We compare TP-GMM-guided RRTConnect against the baseline RRTConnect planner with uniform sampling. This baseline was selected because RRTConnect is the default and most commonly used OMPL planner in MoveIt-based manipulation workflows, and the underlying GMM-informed planner was benchmarked against additional low-level planners in our previous work (Shaarawy et al., 2026). The present evaluation therefore emphasizes the practical system-level question of whether the learned sampling prior improves real dual-arm disassembly under identical perception, task allocation, and reactive collision-monitoring conditions. Adding a broad set of hardware baselines would be valuable, but each dual-arm disassembly trial requires resetting the battery workspace, monitoring two manipulators, and handling safety supervision; such large-scale real-world benchmarking is beyond the scope of this deployment-focused study. Performance is assessed by (i) disassembly time per object and overall makespan (a proxy for coordination efficiency), (ii) end-effector path length (trajectory efficiency), and (iii) swept-volume overlap (spatial footprint in the shared workspace - Equation 11). Both planners execute under the same task allocation and perception pipeline (Section 4.1).

Table 2 summarizes the results of five trials recorded for each planner in one disassembly environment setup. The number of real-world experiments remains limited in this case study as each dual-arm disassembly run is costly, experimentally complex, and time-intensive to record, monitor, and reset. Moreover, given that the disassembly task sequence is fixed by the lookup table (discussed in Section 4.1.2), five trials were considered sufficient to demonstrate the framework’s behavior. Averaged over all six objects, the GMM-informed planner reduces cumulative end-effector path length from 48.8±2.7 m to 17.9±0.9 m (−63.3%) , indicating substantially more compact motions in the shared workspace. It also improves the overall makespan from 467.9±16.4 s to 429.8±37.1 s, despite the added TP-GMM reproduction step. A two-sided Welch t-test on the five trial aggregates confirms that the path-length reduction is statistically significant ( t=24.28 , p<0.001 ), whereas the makespan improvement remains a positive trend but is not significant at this sample size ( t=2.10 , p=0.085 ). This result is consistent with the role of the proposed planner: its strongest measurable benefit is producing more compact, lower-interference motions, while end-to-end cycle time additionally depends on task interleaving and reactive pauses.

**TABLE 2:** Performance comparison of baseline RRTConnect and the GMM-informed planner for multi-robot electric-vehicle battery disassembly. Values are reported as mean ± standard deviation over five trials.

Robot	Object name	Baseline RRTConnect		GMM-informed	
		Time (s)	Path length (m)	Time (s)	Path length (m)
Robot 1	Cable	79.5±19.4	7.0±2.2	86.8±10.6	3.5±0.4
	Busbar	80.7±12.9	8.4±3.2	85.3±21.2	3.1±0.7
	Service plug	75.9±13.1	7.9±0.6	63.3±17.7	2.3±0.1
Robot 2	Battery Module1	76.5±19.9	9.0±2.4	67.9±8.5	3.4±0.8
	Battery Module2	73.1±16.7	8.4±2.3	55.8±5.1	2.4±0.4
	Battery Module3	82.3±18.3	7.9±2.4	70.6±14.2	3.1±0.4
	Disassembly time	467.9±16.4		429.8±37.1	
	Path length		48.8±2.7		17.9±0.9

**FIGURE 11:**
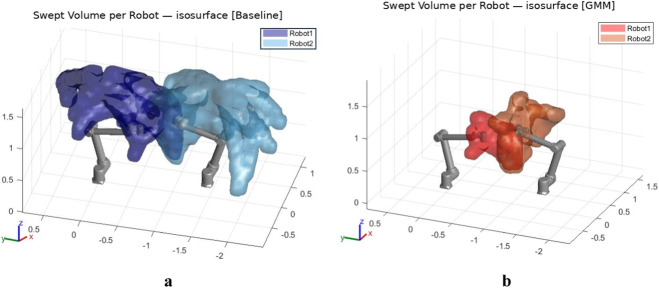
Swept-volume isosurfaces computed on the 0.03 m occupancy grid. **(a)** Baseline RRTConnect and **(b)** the GMM-informed planner.

In terms of per-object trajectory quality, the GMM-informed planner consistently shortens paths across all objects (approximate reductions: Cable ∼50% , Busbar ∼63% , Service Plug ∼71% , Battery Module1 ∼62% , Battery Module2 ∼71% , Battery Module3 ∼61% ). Timing shows complementary strengths: the learning-based planner is faster for the Service Plug and all Battery Module removals ( ≈12 - 24% faster), whereas the baseline planner is slightly faster on Cable and Busbar ( ≈6 - 9% ), where fewer collision-driven detours occur. This behavior reinforces the main contribution of the proposed integration: predictive, demonstration-informed planning is most beneficial when dual-arm interaction and dynamic collision handling matter most. Furthermore, isosurfaces reconstructed from the swept volumes (Figure 11) reveal that the GMM-informed planner constrains the arms to a smaller portion of the workspace and handles collision instances more efficiently. Relative to baseline RRTConnect, the swept volume drops from $V_{1}=0.583\;m^{3}$ to $0.139\;m^{3}$ for Robot 1 (−76%) and from $V_{2}=0.696\;m^{3}$ to $0.252\;m^{3}$ for Robot 2 (−64%) . The mutual overlap volume decreases from $0.064\;m^{3}$ to $0.034\;m^{3}$ (−47%) , showing that the combined predictive/reactive strategy not only shortens paths but also reduces workspace sharing conflicts during concurrent manipulation.

Although the overall results are favourable, several typical failure cases were observed during experimentation. First, the framework remains sensitive to perception outages and occlusions. If a target component becomes temporarily occluded from one or both cameras, or if a camera stream fails during execution, the system may continue searching for the object that is next in the disassembly sequence. In the current implementation, such failures are not always recoverable online and may require restarting the full task. Second, deadlock-like behavior can arise when one robot executes an aggressive collision-avoidance manoeuvre and approaches a near-singular configuration. In this case, the corresponding local planner may become effectively stuck, preventing progress while the other robot waits for the shared workspace to clear. Third, the learned TP-GMM guidance is less reliable when the robot starts from a configuration already close to singularity, because the demonstrated motion priors are not sufficiently representative of those low-dexterity regions. In practice, however, this issue occurs only rarely in our experiments. These observations highlight the need for stronger perception-failure recovery, explicit deadlock-resolution policies, and singularity-aware learning or initialization strategies in future versions of the system.

## 7 Conclusion

This paper presented a perception-driven Task-and-Motion Planning (TAMP) framework for multi-robot EV battery disassembly. The key contribution is the coordination between three essential elements on a real dual-arm platform: (i) task decomposition and task allocation driven by repeated scene perception, (ii) the recently introduced GMM-informed RRT motion planner (Shaarawy et al., 2026), and (iii) a hybrid predictive/reactive collision-handling strategy that combines digital-twin validation with online avoidance and replanning. The challenge is not merely assembling these modules, but making them operate jointly under real-world uncertainty, dual-arm coordination complexity, dynamic obstacles, and simultaneous task-level and geometric constraints.

At the task level, stereo vision with YOLOv8 and depth sensing detects, segments, and localizes components so that logical disassembly constraints can be enforced. An expert-encoded lookup table then drives task decomposition and task allocation while remaining extensible to new components and related sequential tasks. Importantly, the system does not treat task planning and motion planning as fully decoupled: after each action, the robots re-scan the workspace, update completion status, and recompute the remaining task sequence when necessary.

For motion planning, demonstrations are processed into Task-Parameterized Gaussian Mixture Models (TP-GMMs) that guide feasible, expert-like trajectories. Predictive collision checking through OctoMap, MoveIt, and FCL is combined with reactive steering and replanning during execution. Across the three case studies, this integrated formulation yields more compact and safer dual-arm behavior. In the end-to-end disassembly scenario, average path length decreases from 48.8 m to 17.9 m (−63.3%) , with a statistically significant improvement over baseline RRTConnect (p<0.001) , while makespan improves from 467.9 s to 429.8 s (−8.1%) with higher variability across the five trials. Swept-volume analysis further shows substantially smaller per-arm volumes ( R1 : $0.583\to 0.139\;m^{3}$ , R2 : $0.696\to 0.252\;m^{3}$ ) and reduced overlap $\left(0.064\to 0.034\;m^{3}\right)$ , supporting the claim that predictive planning plus reactive collision avoidance improves workspace sharing in real dual-arm disassembly.

Beyond the specific EV-disassembly use case, the modular design of this framework makes it adaptable to larger robot teams and other sequential manipulation tasks. A current limitation is that dual-arm coupling is learned only indirectly through shared scene monitoring and replanning; jointly learned dual-arm motion priors remain an important direction for future work. Overall, the results show that the proposed integration is both practically useful and technically meaningful for coordinated multi-robot manipulation in unstructured and dynamically changing environments.

## Data Availability

The raw data supporting the conclusions of this article will be made available by the authors upon request, without undue reservation.
